# 2-Hy­droxy-7-meth­oxy-9*H*-carbazole-3-carbaldehyde

**DOI:** 10.1107/S1600536810033805

**Published:** 2010-08-28

**Authors:** Hoong-Kun Fun, Wisanu Maneerat, Surat Laphookhieo, Suchada Chantrapromma

**Affiliations:** a X-ray Crystallography Unit, School of Physics, Universiti Sains Malaysia, 11800 USM, Penang, Malaysia; b Natural Products Research Laboratory, School of Science, Mae Fah Luang University, Tasud, Muang, Chiang Rai 57100, Thailand; c Crystal Materials Research Unit, Department of Chemistry, Faculty of Science, Prince of Songkla University, Hat-Yai, Songkhla 90112, Thailand

## Abstract

The title compound, C_14_H_11_NO_3_, was isolated from the roots of *Clausena wallichii*. The carbazole ring system is approx­imately planar (r.m.s. deviation = 0.039 Å) and the dihedral angle between the two benzene rings is 4.63 (7)°. An intra­molecular O—H⋯O hydrogen bond generates an *S*(6) ring motif. In the crystal, mol­ecules are linked into a zigzag network extending parallel to the *ac* plane by O—H⋯N and N—H⋯O hydrogen bonds.

## Related literature

For compounds isolated from plants of genera Rutaceae and their pharmacological activity, see: Ito *et al.* (1997 ▶); Kongkathip & Kongkathip (2009 ▶); Laphookhieo *et al.* (2009 ▶); Li *et al.* (1991 ▶); Maneerat & Laphookhieo (2010 ▶); Maneerat *et al.* (2010 ▶); Sripisut & Laphookhieo (2010 ▶); Tangyuenyongwatthana *et al.* (1992 ▶); Yenjai *et al.* 2000 ▶). For a related structure, see: Fun *et al.* (2009 ▶). For bond-length data, see: Allen *et al.* (1987 ▶). For hydrogen-bond motifs, see: Bernstein *et al.* (1995 ▶). For the stability of the temperature controller used in the data collection, see Cosier & Glazer, (1986 ▶).
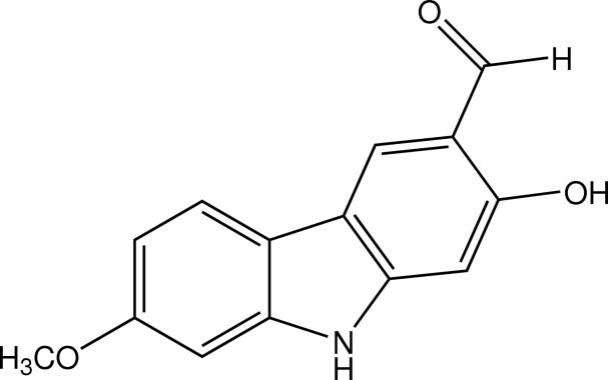


## Experimental

### 

#### Crystal data

C_14_H_11_NO_3_
*M* *_r_* = 241.24
Orthorhombic, 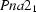

*a* = 12.4352 (4) Å
*b* = 17.6564 (5) Å
*c* = 5.0839 (1) Å
*V* = 1116.23 (5) Å^3^
*Z* = 4
Cu *K*α radiation
μ = 0.84 mm^−1^
*T* = 100 K
0.23 × 0.19 × 0.10 mm

#### Data collection

Bruker APEXII DUO CCD area-detector diffractometer
Absorption correction: multi-scan (*SADABS*; Bruker, 2009 ▶) *T*_min_ = 0.831, *T*_max_ = 0.918
25207 measured reflections
2026 independent reflections
2018 reflections with *I* > 2σ(*I*)
*R*_int_ = 0.027

#### Refinement

*R*[*F* ^2^ > 2σ(*F* ^2^)] = 0.030
*wR*(*F* ^2^) = 0.097
*S* = 1.31
2026 reflections
170 parameters
1 restraint
H atoms treated by a mixture of independent and constrained refinement
Δρ_max_ = 0.64 e Å^−3^
Δρ_min_ = −0.63 e Å^−3^
Absolute structure: Flack (1983 ▶), 851 Friedel pairs
Flack parameter: 0.17 (19)

### 

Data collection: *APEX2* (Bruker, 2009 ▶); cell refinement: *SAINT* (Bruker, 2009 ▶); data reduction: *SAINT*; program(s) used to solve structure: *SHELXTL* (Sheldrick, 2008 ▶); program(s) used to refine structure: *SHELXTL*; molecular graphics: *SHELXTL*; software used to prepare material for publication: *SHELXTL* and *PLATON* (Spek, 2009 ▶).

## Supplementary Material

Crystal structure: contains datablocks global, I. DOI: 10.1107/S1600536810033805/ci5167sup1.cif

Structure factors: contains datablocks I. DOI: 10.1107/S1600536810033805/ci5167Isup2.hkl

Additional supplementary materials:  crystallographic information; 3D view; checkCIF report

## Figures and Tables

**Table 1:** Hydrogen-bond geometry (Å, °)

*D*—H⋯*A*	*D*—H	H⋯*A*	*D*⋯*A*	*D*—H⋯*A*
O1—H1*O*1⋯O2	0.81	1.96	2.6453 (17)	143
O1—H1*O*1⋯N1^i^	0.81	2.53	3.0457 (15)	123
N1—H1*N*1⋯O2^ii^	0.85 (2)	2.10 (2)	2.941 (2)	172 (2)

Symmetry codes: (i) 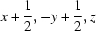; (ii) 
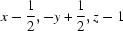
.

**Fig. 1.**
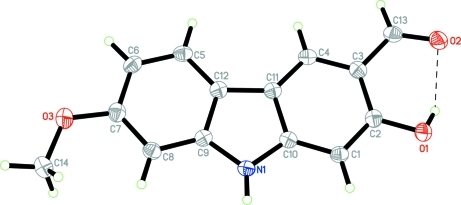
The molecular structure of the title compound, showing 50% probability displacement ellipsoids and the atom-numbering scheme. The O—H···O hydrogen bond is shown as a dashed line.

**Fig. 2.**
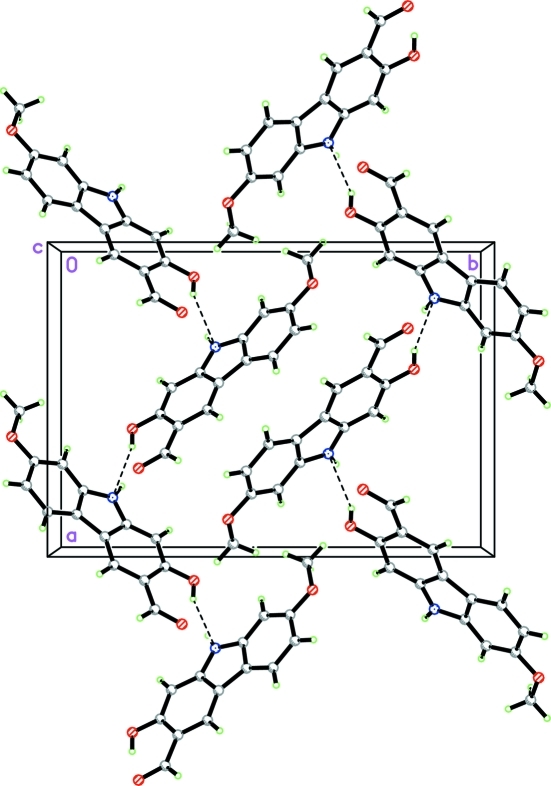
Part of the crystal packing of the title compound, viewed along the c axis. Hydrogen bonds are shown as dashed lines.

## supplementary crystallographic information

### Comment

Rutaceae plants are known to be rich sources of coumarins and carbazole alkaloids. Many of them have been isolated from several genera of Rutaceae especially from *Clausena* genus (Laphookhieo *et al.*, 2009; Maneerat *et al.*, 2010; Sripisut & Laphookhieo 2010; Kongkathip & Kongkathip 2009; Ito *et al.*, 1997; Li *et al.*, 1991; Tangyuenyongwatthana *et al.*, 1992) and some of these compounds show interesting pharmacological activities (Maneerat & Laphookhieo 2010; Yenjai *et al.* 2000). Although *Clausena wallichii* is one of the Rutaceae plants, however phytochemical reports on the chemical constituents from this plant are rare. As part of our continuing study of chemical constituents and bioactive compounds from Thai medicinal plants, we report herein the crystal structure of the title compound, which was isolated from the roots of *C. wallichii* collected from Phrae province in the northern region of Thailand.

The non-hydrogen atoms of the title molecule (Fig. 1) are almost coplanar. The carbazole ring system (C1-C12/N1) is planar with an r.m.s. deviation of 0.039 Å [maximum deviation 0.072 (1) Å for atom C7]. The pyrrole ring makes dihedral angle of 1.66 (7) and 3.12 (8)°, respectively, with the C1–C4/C10–C11 and C5–C9/C12 benzene rings. The dihedral angle between the two benzene rings being 4.63 (7)°. The cabaldehyde and methoxy substituents at atoms C3 and C7, respectively, are coplanar with the benzene ring, as indicated by torsion angles C2–C3–C13–O2 = 0.0 (2)° and C14–O3–C7–C8 = 4.0 (2)°. An intramolecular O1—H1O1···O2 hydrogen bond (Table 1) generates an S(6) ring motif (Fig. 1 and Table 1) (Bernstein *et al.*, 1995). The bond distances are within normal ranges (Allen *et al.*, 1987) and comparable to a related structure (Fun *et al.*, 2009).

The crystal packing of the title compound is stabilized by intermolecular O—H···N and N—H···O hydrogen bonds (Table 1) which link the molecules into a zigzag network extending parallel to the *ac* plane.

### Experimental

The roots of *C. wallichii* (1.02 Kg) were successively extracted with CH_2_Cl_2_ over the period of 3 days each at room temperature to provide the crude CH_2_Cl_2_ extract which subjected to quick column chromatography (QCC) over silica gel eluted with a gradient of hexane-EtOAc (100% hexane to 100% EtOAc) to provide nine fractions (A-I). Fraction G (3.12 g) was further separated by QCC with a gradient of 10% EtOAc-hexane to 100% EtOAc to give seven subfractions (G1-G7). Subfraction G4 (118.9 mg) was subjected to repeated column chromatography using 30% EtOAc-hexane to yield the yellow solid of the title compound (12.8 mg). Yellow plate-shaped single crystals of the title compound suitable for X-ray structure determination were recrystallized from CH_2_Cl_2_/acetone (1:1 v/v) by the slow evaporation of the solvent at room temperature after several days; m.p. 496.7-498.8 K (decomposition).

### Refinement

Atom H1N1 was located in a difference map and refined isotropically. The remaining H atoms were placed in calculated positions with O–H = 0.81, C–H = 0.93 for aromatic and CH, and 0.96 Å for CH_3_ atoms. The *U*_iso_ values were constrained to be 1.5*U*_eq_ of the carrier atom for methyl H atoms and 1.2*U*_eq_ for the remaining H atoms. A rotating group model was used for the methyl groups. The highest residual electron density peak is located at 1.81 Å from C9 and the deepest hole is located at 1.29 Å from C3. 851 Friedel pairs were used to determine the absolute structure.

### Crystal data

**Table:**

C_14_H_11_NO_3_	*D*_x_ = 1.435 Mg m^−^^3^
*M**_r_* = 241.24	Melting point = 496.7–498.8 K
Orthorhombic, *P**n**a*2_1_	Cu *K*α radiation, λ = 1.54178 Å
Hall symbol: P 2c -2n	Cell parameters from 2026 reflections
*a* = 12.4352 (4) Å	θ = 4.4–69.9°
*b* = 17.6564 (5) Å	µ = 0.84 mm^−^^1^
*c* = 5.0839 (1) Å	*T* = 100 K
*V* = 1116.23 (5) Å^3^	Plate, yellow
*Z* = 4	0.23 × 0.19 × 0.10 mm
*F*(000) = 504	

### Data collection

**Table:**

Bruker APEXII DUO CCD area-detector diffractometer	2026 independent reflections
Radiation source: sealed tube	2018 reflections with *I* > 2σ(*I*)
graphite	*R*_int_ = 0.027
φ and ω scans	θ_max_ = 69.9°, θ_min_ = 4.4°
Absorption correction: multi-scan (*SADABS*; Bruker, 2009)	*h* = −14→14
*T*_min_ = 0.831, *T*_max_ = 0.918	*k* = −21→21
25207 measured reflections	*l* = −5→6

### Refinement

**Table:**

Refinement on *F*^2^	Hydrogen site location: inferred from neighbouring sites
Least-squares matrix: full	H atoms treated by a mixture of independent and constrained refinement
*R*[*F*^2^ > 2σ(*F*^2^)] = 0.030	*w* = 1/[σ^2^(*F*_o_^2^) + (0.0656*P*)^2^ + 0.054*P*] where *P* = (*F*_o_^2^ + 2*F*_c_^2^)/3
*wR*(*F*^2^) = 0.097	(Δ/σ)_max_ = 0.001
*S* = 1.31	Δρ_max_ = 0.64 e Å^−^^3^
2026 reflections	Δρ_min_ = −0.63 e Å^−^^3^
170 parameters	Extinction correction: *SHELXTL* (Sheldrick, 2008), Fc^*^=kFc[1+0.001xFc^2^λ^3^/sin(2θ)]^-1/4^
1 restraint	Extinction coefficient: 0.053 (3)
Primary atom site location: structure-invariant direct methods	Absolute structure: Flack (1983), 851 Friedel pairs
Secondary atom site location: difference Fourier map	Flack parameter: 0.17 (19)

### Special details

**Table:**

Experimental. The crystal was placed in the cold stream of an Oxford Cryosystems Cobra open-flow nitrogen cryostat (Cosier & Glazer, 1986) operating at 100.0 (1) K.
Geometry. All esds (except the esd in the dihedral angle between two l.s. planes) are estimated using the full covariance matrix. The cell esds are taken into account individually in the estimation of esds in distances, angles and torsion angles; correlations between esds in cell parameters are only used when they are defined by crystal symmetry. An approximate (isotropic) treatment of cell esds is used for estimating esds involving l.s. planes.
Refinement. Refinement of F^2^ against ALL reflections. The weighted R-factor wR and goodness of fit S are based on F^2^, conventional R-factors R are based on F, with F set to zero for negative F^2^. The threshold expression of F^2^ > 2sigma(F^2^) is used only for calculating R-factors(gt) etc. and is not relevant to the choice of reflections for refinement. R-factors based on F^2^ are statistically about twice as large as those based on F, and R- factors based on ALL data will be even larger.

### Fractional atomic coordinates and isotropic or equivalent isotropic displacement parameters (Å^2^)

**Table:**

	*x*	*y*	*z*	*U*_iso_*/*U*_eq_	
O1	0.59667 (8)	0.17861 (6)	0.4400 (2)	0.0264 (3)	
H1O1	0.6537	0.1796	0.5149	0.056 (7)*	
O2	0.72058 (8)	0.20104 (6)	0.8557 (3)	0.0294 (3)	
O3	0.12071 (9)	0.59553 (6)	0.5822 (3)	0.0325 (3)	
N1	0.32559 (9)	0.37021 (7)	0.3111 (3)	0.0203 (3)	
H1N1	0.2898 (14)	0.3514 (10)	0.185 (5)	0.029 (5)*	
C1	0.46098 (10)	0.26763 (8)	0.3568 (3)	0.0213 (3)	
H1A	0.4366	0.2391	0.2150	0.026*	
C2	0.54764 (10)	0.24453 (8)	0.5070 (3)	0.0212 (3)	
C3	0.58509 (11)	0.28796 (8)	0.7249 (3)	0.0220 (3)	
C4	0.53291 (10)	0.35607 (8)	0.7921 (3)	0.0208 (3)	
H4A	0.5569	0.3847	0.9339	0.025*	
C5	0.36091 (11)	0.50464 (7)	0.8333 (3)	0.0226 (3)	
H5A	0.4092	0.5117	0.9708	0.027*	
C6	0.27613 (11)	0.55440 (8)	0.7967 (3)	0.0241 (3)	
H6A	0.2680	0.5955	0.9094	0.029*	
C7	0.20217 (11)	0.54348 (8)	0.5906 (3)	0.0238 (3)	
C8	0.21259 (10)	0.48413 (7)	0.4134 (3)	0.0222 (4)	
H8A	0.1644	0.4774	0.2756	0.027*	
C9	0.29943 (10)	0.43496 (7)	0.4528 (3)	0.0195 (3)	
C10	0.41167 (10)	0.33567 (7)	0.4277 (3)	0.0196 (3)	
C11	0.44591 (10)	0.37986 (8)	0.6456 (3)	0.0194 (3)	
C12	0.37263 (11)	0.44372 (8)	0.6603 (3)	0.0203 (3)	
C13	0.67188 (10)	0.26163 (8)	0.8877 (3)	0.0244 (3)	
H13A	0.6930	0.2923	1.0273	0.029*	
C14	0.03861 (12)	0.58496 (9)	0.3899 (4)	0.0354 (4)	
H14A	−0.0179	0.6211	0.4184	0.053*	
H14B	0.0101	0.5346	0.4043	0.053*	
H14C	0.0684	0.5921	0.2174	0.053*	

### Atomic displacement parameters (Å^2^)

**Table:**

	*U*^11^	*U*^22^	*U*^33^	*U*^12^	*U*^13^	*U*^23^
O1	0.0261 (5)	0.0281 (5)	0.0251 (6)	0.0080 (4)	−0.0009 (4)	−0.0024 (5)
O2	0.0282 (5)	0.0358 (6)	0.0241 (7)	0.0098 (4)	−0.0016 (4)	0.0011 (5)
O3	0.0345 (6)	0.0291 (6)	0.0340 (7)	0.0104 (4)	−0.0052 (5)	−0.0053 (5)
N1	0.0207 (6)	0.0215 (6)	0.0187 (6)	0.0000 (4)	−0.0025 (5)	−0.0007 (5)
C1	0.0224 (6)	0.0225 (7)	0.0188 (8)	−0.0013 (5)	0.0010 (6)	−0.0003 (5)
C2	0.0202 (6)	0.0226 (6)	0.0209 (8)	0.0007 (5)	0.0047 (5)	0.0017 (6)
C3	0.0206 (7)	0.0242 (7)	0.0212 (8)	−0.0011 (5)	0.0019 (6)	0.0032 (6)
C4	0.0207 (6)	0.0234 (7)	0.0183 (8)	−0.0035 (5)	0.0000 (5)	0.0005 (6)
C5	0.0257 (7)	0.0221 (7)	0.0199 (7)	−0.0044 (5)	−0.0007 (6)	0.0000 (6)
C6	0.0303 (7)	0.0204 (6)	0.0217 (8)	−0.0017 (5)	0.0022 (6)	−0.0028 (6)
C7	0.0258 (7)	0.0210 (7)	0.0246 (8)	0.0016 (5)	0.0025 (6)	0.0029 (6)
C8	0.0226 (7)	0.0224 (7)	0.0216 (9)	−0.0004 (5)	−0.0014 (5)	0.0023 (6)
C9	0.0201 (6)	0.0195 (6)	0.0191 (8)	−0.0028 (5)	0.0020 (5)	0.0005 (6)
C10	0.0179 (6)	0.0217 (7)	0.0193 (8)	−0.0026 (4)	0.0014 (5)	0.0020 (6)
C11	0.0211 (6)	0.0191 (6)	0.0180 (8)	−0.0036 (5)	0.0023 (5)	0.0016 (5)
C12	0.0214 (7)	0.0188 (6)	0.0206 (8)	−0.0030 (5)	0.0015 (5)	0.0031 (6)
C13	0.0238 (6)	0.0297 (7)	0.0198 (9)	−0.0003 (5)	−0.0001 (6)	0.0025 (6)
C14	0.0353 (8)	0.0360 (9)	0.0348 (11)	0.0131 (6)	−0.0077 (7)	−0.0022 (8)

### Geometric parameters (Å, °)

**Table:**

O1—C2	1.3574 (17)	C5—C6	1.3850 (19)
O1—H1O1	0.81	C5—C12	1.397 (2)
O2—C13	1.2401 (17)	C5—H5A	0.93
O3—C7	1.3685 (16)	C6—C7	1.407 (2)
O3—C14	1.426 (2)	C6—H6A	0.93
N1—C10	1.3671 (18)	C7—C8	1.388 (2)
N1—C9	1.3899 (18)	C8—C9	1.4000 (17)
N1—H1N1	0.85 (2)	C8—H8A	0.93
C1—C2	1.382 (2)	C9—C12	1.402 (2)
C1—C10	1.3960 (18)	C10—C11	1.420 (2)
C1—H1A	0.93	C11—C12	1.4517 (18)
C2—C3	1.425 (2)	C13—H13A	0.93
C3—C4	1.4086 (19)	C14—H14A	0.96
C3—C13	1.438 (2)	C14—H14B	0.96
C4—C11	1.379 (2)	C14—H14C	0.96
C4—H4A	0.93		
			
C2—O1—H1O1	104.9	C8—C7—C6	121.71 (13)
C7—O3—C14	117.62 (13)	C7—C8—C9	116.58 (13)
C10—N1—C9	109.00 (13)	C7—C8—H8A	121.7
C10—N1—H1N1	124.3 (12)	C9—C8—H8A	121.7
C9—N1—H1N1	126.2 (12)	N1—C9—C8	128.06 (14)
C2—C1—C10	116.99 (14)	N1—C9—C12	109.20 (12)
C2—C1—H1A	121.5	C8—C9—C12	122.69 (13)
C10—C1—H1A	121.5	N1—C10—C1	128.02 (14)
O1—C2—C1	117.68 (14)	N1—C10—C11	109.13 (12)
O1—C2—C3	120.64 (13)	C1—C10—C11	122.84 (13)
C1—C2—C3	121.67 (13)	C4—C11—C10	119.28 (13)
C4—C3—C2	119.83 (13)	C4—C11—C12	134.53 (14)
C4—C3—C13	118.83 (14)	C10—C11—C12	106.18 (12)
C2—C3—C13	121.25 (13)	C5—C12—C9	119.41 (12)
C11—C4—C3	119.36 (14)	C5—C12—C11	134.10 (14)
C11—C4—H4A	120.3	C9—C12—C11	106.45 (12)
C3—C4—H4A	120.3	O2—C13—C3	124.75 (14)
C6—C5—C12	118.88 (14)	O2—C13—H13A	117.6
C6—C5—H5A	120.6	C3—C13—H13A	117.6
C12—C5—H5A	120.6	O3—C14—H14A	109.5
C5—C6—C7	120.71 (14)	O3—C14—H14B	109.5
C5—C6—H6A	119.6	H14A—C14—H14B	109.5
C7—C6—H6A	119.6	O3—C14—H14C	109.5
O3—C7—C8	123.77 (14)	H14A—C14—H14C	109.5
O3—C7—C6	114.52 (14)	H14B—C14—H14C	109.5
			
C10—C1—C2—O1	−179.79 (13)	C2—C1—C10—N1	179.92 (13)
C10—C1—C2—C3	0.13 (19)	C2—C1—C10—C11	−1.0 (2)
O1—C2—C3—C4	−179.74 (12)	C3—C4—C11—C10	−0.9 (2)
C1—C2—C3—C4	0.3 (2)	C3—C4—C11—C12	177.97 (14)
O1—C2—C3—C13	−3.2 (2)	N1—C10—C11—C4	−179.38 (12)
C1—C2—C3—C13	176.88 (12)	C1—C10—C11—C4	1.4 (2)
C2—C3—C4—C11	0.0 (2)	N1—C10—C11—C12	1.49 (15)
C13—C3—C4—C11	−176.59 (13)	C1—C10—C11—C12	−177.74 (12)
C12—C5—C6—C7	0.7 (2)	C6—C5—C12—C9	0.8 (2)
C14—O3—C7—C8	4.0 (2)	C6—C5—C12—C11	−176.30 (14)
C14—O3—C7—C6	−175.73 (14)	N1—C9—C12—C5	−178.86 (12)
C5—C6—C7—O3	178.13 (13)	C8—C9—C12—C5	−1.4 (2)
C5—C6—C7—C8	−1.7 (2)	N1—C9—C12—C11	−1.06 (15)
O3—C7—C8—C9	−178.74 (13)	C8—C9—C12—C11	176.40 (12)
C6—C7—C8—C9	1.0 (2)	C4—C11—C12—C5	−1.9 (3)
C10—N1—C9—C8	−175.26 (13)	C10—C11—C12—C5	177.08 (15)
C10—N1—C9—C12	2.04 (15)	C4—C11—C12—C9	−179.18 (15)
C7—C8—C9—N1	177.45 (14)	C10—C11—C12—C9	−0.25 (15)
C7—C8—C9—C12	0.49 (19)	C4—C3—C13—O2	176.58 (13)
C9—N1—C10—C1	176.99 (13)	C2—C3—C13—O2	0.0 (2)
C9—N1—C10—C11	−2.19 (15)		

### Hydrogen-bond geometry (Å, °)

**Table:**

*D*—H···*A*	*D*—H	H···*A*	*D*···*A*	*D*—H···*A*
O1—H1O1···O2	0.81	1.96	2.6453 (17)	143
O1—H1O1···N1^i^	0.81	2.53	3.0457 (15)	123
N1—H1N1···O2^ii^	0.85 (2)	2.10 (2)	2.941 (2)	172 (2)

Symmetry codes:  (i) *x*+1/2, −*y*+1/2, *z*; (ii) *x*−1/2, −*y*+1/2, *z*−1.
